# A novel ferroptosis-related gene signature can predict prognosis and influence immune microenvironment in acute myeloid leukemia

**DOI:** 10.17305/bjbms.2021.6274

**Published:** 2021-11-16

**Authors:** Xianbo Huang, De Zhou, Xiujin Ye, Jie Jin

**Affiliations:** Department of Hematology, The First Affiliated Hospital, College of Medicine, Zhejiang University, Hangzhou, China

**Keywords:** Acute myeloid leukemia, ferroptosis, prognostic gene signature, overall survival, tumor immune microenvironment, drug resistance

## Abstract

Acute myeloid leukemia (AML) is a highly heterogeneous hematopoietic malignancy that strongly correlates with poor clinical outcomes. Ferroptosis is an iron-dependent, non-apoptotic form of regulated cell death which plays an important role in various human cancers. Nevertheless, the prognostic significance and functions of ferroptosis-related genes (FRGs) in AML have not received sufficient attention. The aim of this article was to evaluate the association between FRGs levels and AML prognosis using publicly available RNA-sequencing datasets. The univariate Cox regression analysis identified 20 FRGs that correlate with patient overall survival (OS). The LASSO Cox regression model was used to construct a prognostic 12-gene risk model using a TCGA cohort, and internal and external validation proved the signature efficient. The 12 FRGs signature was then used to assign patients into high- and low-risk groups, with the former exhibiting markedly reduced OS, compared to the low-risk group. ROC curve analysis verified the predictive ability of the risk model. Functional analysis showed that immune status and drug sensitivity differed between the two risk groups. In summary, FRGs is a promising candidate biomarker and therapeutic target for AML.

## INTRODUCTION

Acute myeloid leukemia (AML), the most prevalent leukemia in adults, is a heterogeneous malignancy that arises from clonal expansion of transformed pluripotent hematopoietic stem cells. It is associated with various genomic alterations [1,2]. Efforts have been aimed at elucidating the genetic landscape of AML, however, the current therapeutic options, including allogeneic hematopoietic stem cell transplantation and intensive chemotherapy, have not led to significant improvements in clinical outcomes [3,4]. Majority of AML patients suffer relapse while overall survival (OS) rate remains <30% [4]. Thus, a better understanding of the molecular basis of AML may identify novel diagnostic and prognostic biomarkers of AML, as well as therapeutic targets for better outcomes.

Apoptosis induction by chemotherapy is a key mechanism underlying leukemia cell death [5]. However, relapsed/refractory AML is often resistant to apoptosis [6,7], calling for novel ways of inducing cell death. Ferroptosis, a form of regulated cell death (RCD), has recently been reported. It is iron dependent and differs from apoptosis, autophagy, necrosis, necroptosis, pyroptosis, and other cell death forms [8]. It is defined by cell membrane ruptures and blisters, shrinkage of the mitochondrial, enhanced membrane density, decrease or vanishing of mitochondrial ridges, outer mitochondrial membrane rupture, and nuclei that are normal sized but lacking condensed chromatin. Ferroptosis is triggered by accumulation of iron, excess reactive oxygen species (ROS) levels, and high lipid peroxidation levels [9,10]. A set of genes, which are associated with various metabolic changes, have been shown to regulate ferroptosis. Several pathways, including mevalonate and iron, lipid, as well as glucose metabolism, are involved in ferroptosis. Glutathione peroxidase 4 (GPX4) and cystine-glutamate antiporter (system XC-) are crucial ferroptosis pathway components. Iron uptake and use are vital for ferroptosis [11,12].

Dysregulation of ferroptosis is observed in a wide range of disorders, including cancer. Ferroptosis induction is a likely therapeutic avenue for triggering cancer cell death, particularly for tumors with resistance to traditional therapies [13,14]. Ferroptosis has important roles in AML. Upregulation of GPX4, a phospholipid (PL) hydroperoxidase that negatively regulates ferroptosis, correlates with poor AML prognosis [15]. Aldehyde dehydrogenase 3a2, a long-chain aliphatic aldehyde-oxidizing enzyme, protects AML cells from oxidative death and is highly lethal with GPX4 suppression-mediated ferroptosis [16]. APR-245 (p53-mutated protein inhibitor) [17], ATPR (a novel all-trans retinoic acid derivative) [18], FTY720 (sphingosine-1-phosphate inhibitor) [19], typhaneoside [20], and dihydroartemisinin (DHC) [21] can induce ferroptosis in AML cells. However, the molecular basis of ferroptosis and its role in AML prognosis is unclear.

Here, we elucidate on the features of ferroptosis-related genes (FRGs) in AML using publicly available AML RNA-seq data and the equivalent clinical data. We then constructed a prognostic 12 FRGs risk signature using the TCGA cohort and validated it on GEO datasets. Functional analyses were conducted to elucidate on the pathomechanisms. Our findings show the ferroptosis prognostic landscape, which has potential application in predicting AML prognosis.

## MATERIALS AND METHODS

### Data collection from publicly available databases

RNA-seq data belonging to 151 AML patients were obtained from TCGA (https://portal.gdc.cancer.gov/). After exclusion of samples that lacked corresponding clinical data, data on 130 AML samples remained for analysis. In addition, the Series Matrix File of dataset GSE71014 was retrieved from GEO (https://www.ncbi.nlm.nih.gov/geo/). Then, the platform’s gene probes were transmuted into gene names through referencing the GPL10588 platform. Next, data on 104 AML patients who had complete gene expression profiles as well as survival data were retrieved. The Series Matrix File for dataset GSE12417 (annotation platform: GPL570) containing data on 78 AML patients who had complete gene expression profiles as well as survival data was also retrieved. A total of 103 FRGs were retrieved from GeneCards (https://www.genecards.org/) and BioGPS (http://www.biogps.org/). We then extracted the shared FRGs from the three datasets (TCGA-LAML, GSE71014, and GSE12417) and excluded poorly expressed genes whose value was “0” in over half of the samples, or had “<0.3” as the mean value of expression.

### Functional annotation of AML-related FRGs

Survival analysis was conducted on AML-related FRGs. Univariate Cox evaluation of OS was done to establish FRGs with prognostic value, with *p* < 0.05 indicating statistical significance. Functional analysis of the AML-related FRGs using gene ontology (GO) and Kyoto Encyclopedia of Genes and Genomes (KEGG) analyses was done through “clusterProfiler” in R [22]. Cutoff *p*- and *p*-values were set at 0.05.

### Establishment and verification of a prognostic FRGs signature

To minimize overfitting risk, least absolute shrinkage and selection operator (LASSO)-penalized Cox regression analyses were conducted to establish candidate genes for use in the risk score signature. Risk features for prognosis were evaluated using “glmnet” and “survival” packages on R using LASSO [23]. We used a k-fold (*k* = 10) cross-validation in the LASSO regression. The dataset was randomly divided into 10 individual subjects, and the ratio of training and validation sets was 9:1. We trained the models with k−1(9) folds and validated them with the rest one. In the regression analysis, the normalized expression matrix of candidate prognostic FRGs was used as the independent variable. AML OS for the TCGA cohort was the response variable. Finally, 12 candidate FRGs were selected and entered further analysis. Calculation of AML patients’ AML risk scores was based on normalized levels for every FRG and its regression coefficients using the formula: Score = sum (corresponding coefficient × each gene’s expression). The median risk score was used to stratify patients into low- and high-risk groups. Two-sided log-rank tests and Kaplan–Meier survival analyses were performed to determine differences in OS between the two groups. LASSO analysis was used to test the risk score’s capacity for prognostic prediction. Receiver operating characteristic (ROC) curve analysis assessed the model’s prognostic accuracy.

### Prediction of clinical chemotherapeutic response

Next, we used the “pRRophetic” [24] package on R to predict clinical chemotherapeutic response for every AML patient in the above cohort based on the Genomics of Drug Sensitivity in Cancer (GDSC) database (https://www.cancerrxgene.org/). The projected IC_50_ for every cell line exposed to a particular drug was acquired through ridge regression, while predictive accuracies were determined through 10-fold cross-validation using the GDSC training set. For all parameters, including “combat,” default values were obtained for removal of batch effect and mean value for summarizing duplicate gene expression [25].

### Evaluation of tumor-infiltrating immune cells (TIICs)

CIBERSORT (https://cibersort.stanford.edu/) is an algorithm for analyzing RNA expression data and determining the proportions of various cell subtypes in every sample [26]. Next, we used CIBERSORT to determine the proportion of TIICs in the subgroups of AML samples. CIBERSORT offers R language computing source code and LM22 signature gene matrix [26]. The R programs comprise “preprocessCore” and “e1071” packages. In the R program, 1000 (recommended value is >100) was set as the statistical rank while quantile normalization was disabled. Next, the ratios for relative infiltrations of 22 distinct immune cell types were evaluated, with 1 as the sum of fractions. Spearman correlation analysis using “cor.test” in R was used to evaluate associations between infiltrations of immune cells and gene expressions. *p* < 0.05 was the cutoff for significance.

### Gene set enrichment analysis (GSEA)

GSEA (https://www.broadlnstitute.org/gsea/) was used to identify differential expressions of genes (gene sets) that were functionally related and whose enrichment in AML patient subgroups was significant [27]. GO and KEGG pathway databases, which were used as functional enrichment reference sets, were obtained from the Molecular Signatures Database [28]. Minimal and maximal gene set sizes were set at 20 and 500, respectively. GSEA performed 1000 permutation, and gene sets with *p* < 0.05 and false discovery rate ≤0.25 were significant. Finally, markedly enriched KEGG pathways and GO terms are concentratedly displayed.

### Tumor-immune interaction analysis

Tumor-Immune System Interactions Database (TISIDB; http://cis.hku.hk/TISIDB/index.php) is an integrated repository of data from several databases, including TCGA, UniProt, DrugBank, and GO, and is used to assess tumor-immune associations across human cancers [29]. We used TISIDB to assess the relationship between TIIC-related signatures and expression levels of FRGs included in the prognostic risk score model using Spearman’s test. *p* < 0.05 was the cutoff for significance and all tests were two sided. These findings were presented on heatmaps developed using “pheatmap” in R.

### Statistical analysis

Analyses were done using R v3.6 (www.r-project.org). Based on the FRGs signature, two-sided log-rank tests and Kaplan–Meier survival analyses were conducted to determine OS differences between high- and low-risk group patients. A Cox proportional hazards regression model was used for multivariate analysis. ROC curve analysis using “pROC”in R was used for OS prediction. Associations between clinical variants and the risk score were evaluated by the Chi-square test. *p* < 0.05 denoted significance.

## RESULTS

**FIGURE 1:**
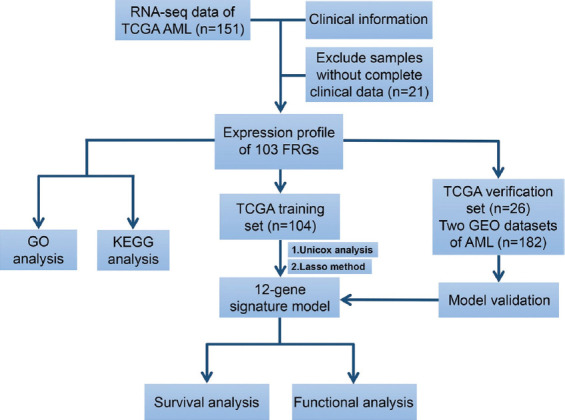
Schematic presentation of this study.

Figure 1 shows the schematic presentation of this study. One hundred and thirty AML patients from the TCGA-LAML cohort were recruited in this study and their clinical features are shown in Table 1.

**TABLE 1:**
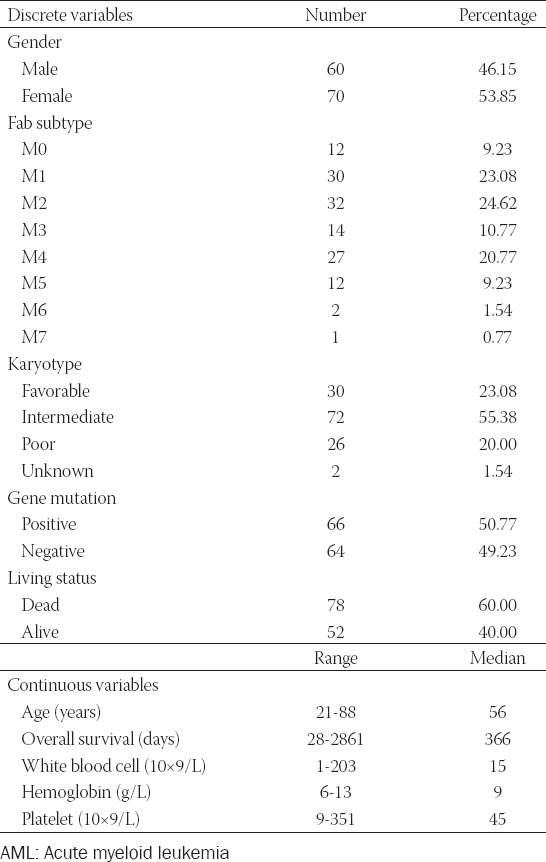
Clinical characteristics of AML patients in TCGA cohort

### Prognostic FRGs in AML patients

**FIGURE 2:**
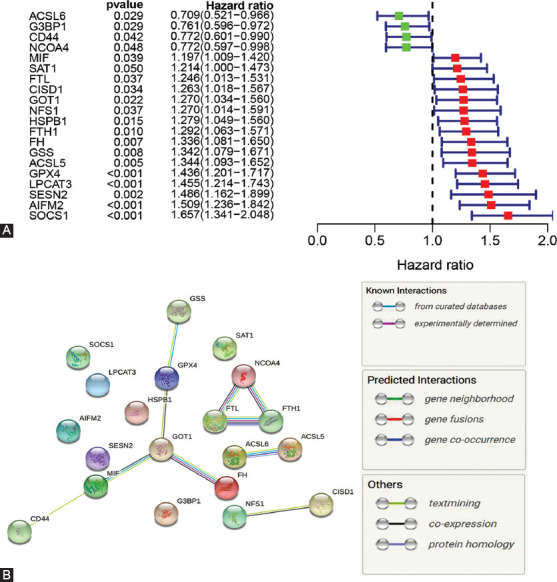
Identification of prognostic FRGs in TCGA AML cohort. (A) Forest plot with hazard ratios of the univariate Cox proportional hazards regression analysis between FRGs expression and OS. (B) PPI network for interactions among the 20 FRGs. FRGs: Ferroptosis-related genes, AML: Acute myeloid leukemia, OS: Overall survival.

Gene expression data for the AML cohort were retrieved from TCGA as well as FRGs expression patterns extracted from the dataset. On exclusion of poorly expressed genes with a value of “0” in over half the samples, or <0.3 as the mean expression value, 94 FRGs remained for analysis (Table S1). Univariate Cox regression survival analysis on the 94 FRGs identified 20 that significantly correlated with AML prognosis (all *p* < 0.05, Figure 2A). Interactions among these genes are presented in Figure 2B. Detailed information on the 20 AML-related FRGs is shown on Table S2.

### Functional analysis of the AML-associated FRGs

**FIGURE 3:**
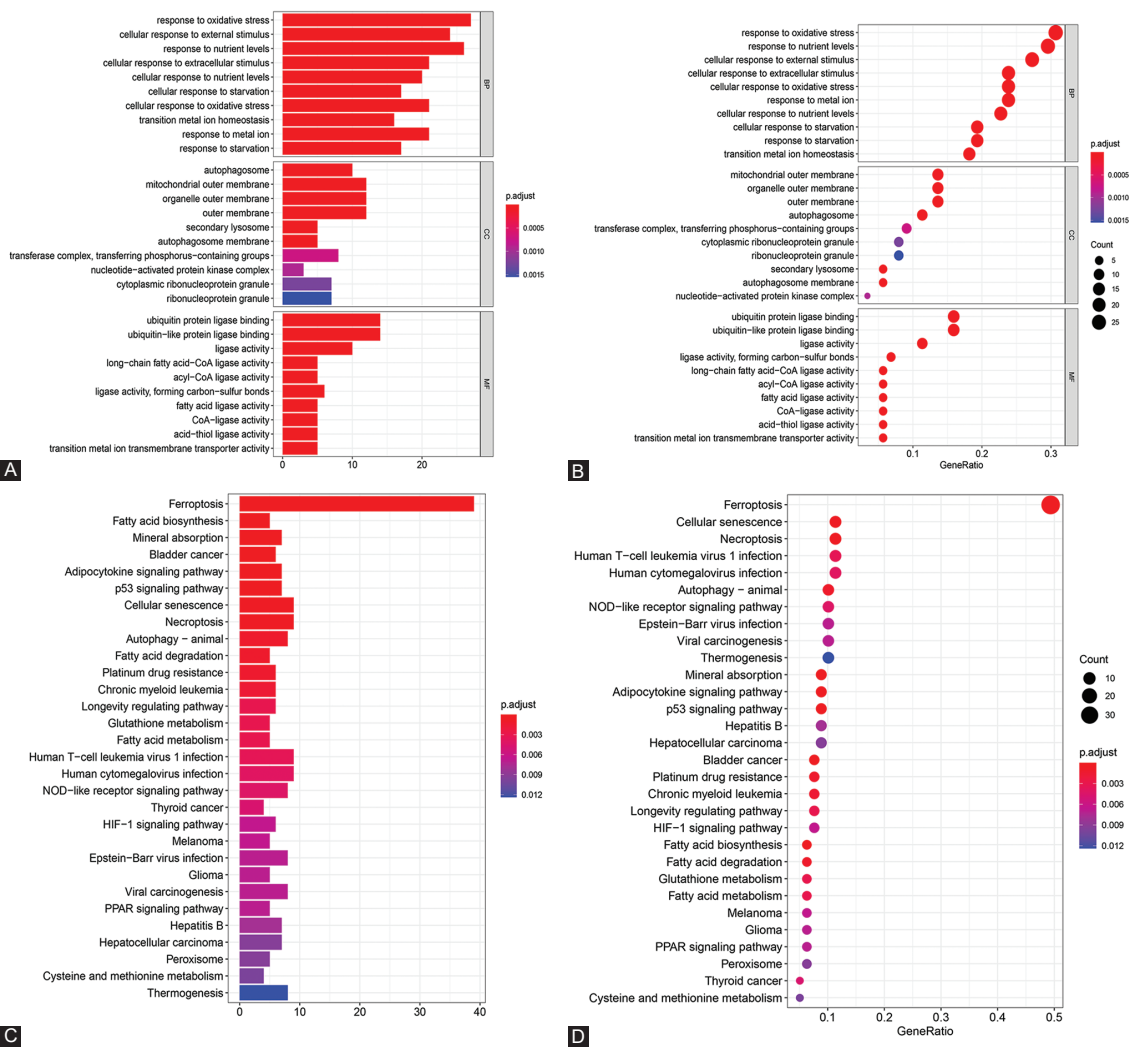
GO and KEGG analyses of AML-associated FRGs. (A and B) GO enrichment. (C and D) KEGG pathway enrichment. GO: Gene ontology, KEGG: Kyoto encyclopedia of genes and genomes, FRGs: Ferroptosis-related genes, AML: Acute myeloid leukemia.

For the 20 FRGs, GO analysis of biological processes (BP) found that these genes were most enriched in cell responses to external stimulus, oxidative stress responses, and nutrient level responses. With regard to cellular component, these genes were highly enriched in the outer membrane of the mitochondria, outer membrane of organelles, and in autophagosomes. In terms of molecular function, these genes were highly enriched in ubiquitin-like protein ligase binding and ubiquitin protein ligase binding (Figure 3A and B). KEGG pathway analysis (Figure 3C and D) revealed that AML-associated FRGs were enriched in various pathways, especially ferroptosis, fatty acid biosynthesis, mineral absorption, adipocytokine signaling pathway, and p53 signaling.

### Prognostic gene signatures associated with AML and ferroptosis

**FIGURE 4:**
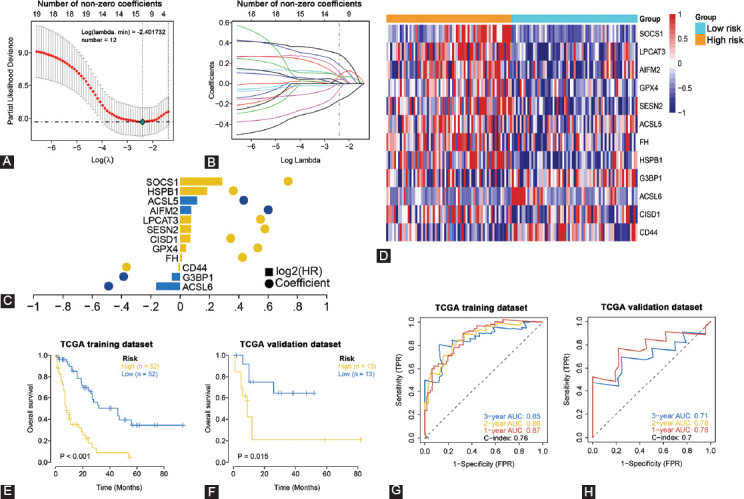
Establishment of a 12 FRGs risk signature for OS by LASSO regression analysis in the TCGA cohort. (A) Ten-fold cross-validation for tuning parameter (lambda) selection in the LASSO model. (B) The LASSO coefficient profile plot was created against the log (lambda) sequence. (C) Results of 12 selected FRGs and their regression coefficients by LASSO. (D) Heatmap of the levels of the 12 FRGs in high- and low-risk score groups. (E and F) Kaplan–Meier curve analysis of AML patients’ OS stratified by risk score in the TCGA training and validation sets. (G and H) Time-dependent ROC curves in the TCGA training and validation sets. FRGs: Ferroptosis-related genes, OS: Overall survival, LASSO: Least absolute shrinkage and selection operator, ROC: Receiver operating characteristic, AML: Acute myeloid leukemia.

To assess the prognostic value of FRGs in AML, the TCGA AML cohort was randomized into verification and training sets at a 1:4 ratio and LASSO Cox regression analysis used to develop a prognostic model based on expression levels of the 20 FRGs, generating a 12-gene signature (Figure 4A-C and Table S3). Patients’ risk scores were evaluated from regression coefficients and expression levels. Risk score signatures were evaluated through the formula: Risk score = (−0.164270341) * acyl-CoA synthetase long-chain family (ACSL)6 levels + (−0.05745754) * Ras-GTPase-activating protein-binding protein 1 (G3BP1) levels + (−0.012600572) * CD44 levels + 0.011883347 * FH levels + 0.040188616 * GPX4 levels + 0.070266332 * CISD1 levels + 0.073905513 * SESN2 levels + 0.074774143 * LPCAT3 levels + 0.075162582 * AIFM2 levels + 0.115640755 * ACSL5 levels + 0.184767081 * HSPB1 levels + 0.287328369 * SOCS1 levels.

To evaluate the signatures’ performance, patients were stratified into a low- and high-risk group based on median cutoff values and expressions of the 12 FRGs shown on a heatmap (Figure 4D). Kaplan–Meier analysis revealed that relative to low-risk patient group, in both training set and verification set, the high-risk AML patient group exhibited markedly worse OS (Figure 4E and F). From ROC curves, risk score signature AUC values of 0.87 (1 year), 0.86 (2 years), and 0.85 (3 years) in the training set as well as a C-index of 0.76 were obtained (Figure 4G). In the validation set, the AUC values were 0.78 for 1 year, 0.78 for 2 years, and 0.71 for 3 years and a C-index of 0.7 (Figure 4H). The results implied that the FRGs signature exhibited a modest AML survival predictive power.

### Validation of the 12 FRGs signature on independent cohorts

**FIGURE 5:**
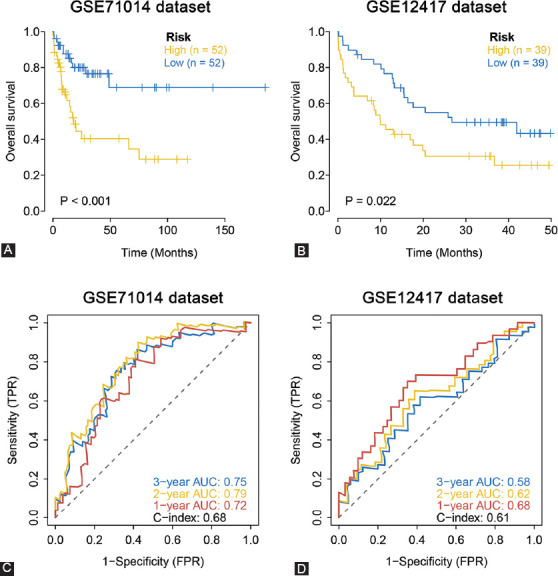
External validation of the 12 FRGs signature in the GEO cohort. (A and B) Kaplan–Meier curves for the OS of patients in different risk groups of two GEO external validation datasets. (C and D) AUC of time-dependent ROC curves in the two GEO.

GEO datasets GSE71014 and GSE12417 were independent cohorts for external validation. AML patients in the GEO dataset were grouped into high- or low-risk patient groups with regard to the median risk score obtained as calculated for the TCGA dataset. Kaplan–Meier analysis results confirmed the signature’s prognostic ability, with high-risk patients exhibiting low OS relative to low-risk patient groups in GSE71014 and GSE12417 (Figure 5A and B). In the GSE71014 dataset, AUC values for the risk score signature were 0.72 (1 year), 0.79 (2 years), and 0.75 (3 years), with a C-index of 0.68 (Figure 5C). In GSE12417 dataset, AUC values were 0.68 for 1 year, 0.62 for 2 years, and 0.58 for 3 years with a C-index of 0.61 (Figure 5D). Our data strongly confirmed the 12 FRGs signature’s high prognostic capacity for AML.

### Functional analysis in high- and low-risk patient groups using GSEA

**FIGURE 6:**
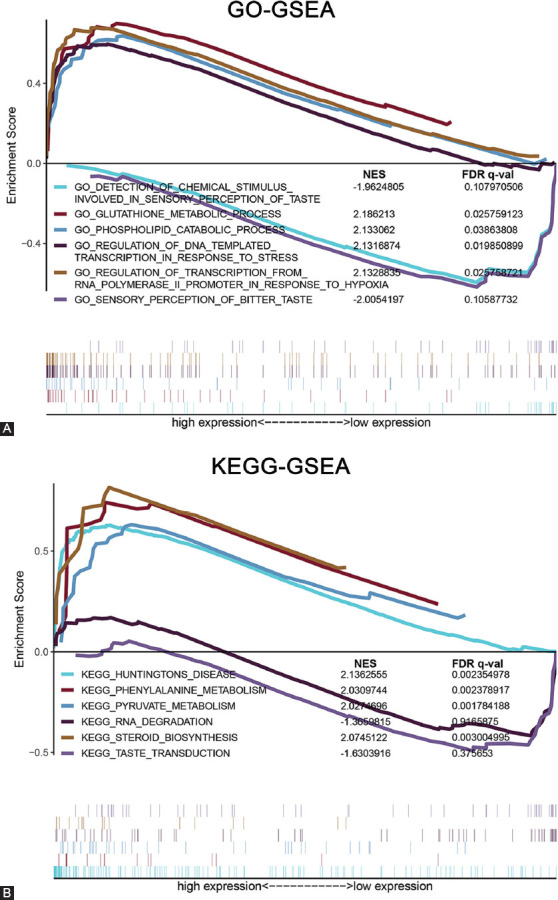
GSEA analysis of DEGs between low- and high-risk AML subgroups. (A) GSEA GO term enrichment. (B) GSEA KEGG pathway enrichment. GSEA: Gene set enrichment analysis, DEGs: Differentially expressed genes, AML: Acute myeloid leukemia, GO: Gene ontology, KEGG: Kyoto encyclopedia of genes and genomes.

To establish risk score-associated biological functions and pathways, we subjected differentially expressed genes (DEGs) in high- versus low-risk AML groups in the TCGA cohort to GSEA-based GO and KEGG analysis. GO-GSEA results showed that several biological functions were alternated in patients with high-risk scores, such as glutathione metabolic process and PL catabolic process (Figure 6A). KEGG-GSEA analysis showed that several pathways, including phenylalanine metabolism and pyruvate metabolism, were markedly enriched in the high-risk patient group (Figure 6B). Taken together, these findings indicate that by affecting these cellular processes as well as pathways, DEGs in high-risk groups cause poor AML prognosis.

### Immune correlations with the 12 FRGs signature

**FIGURE 7:**
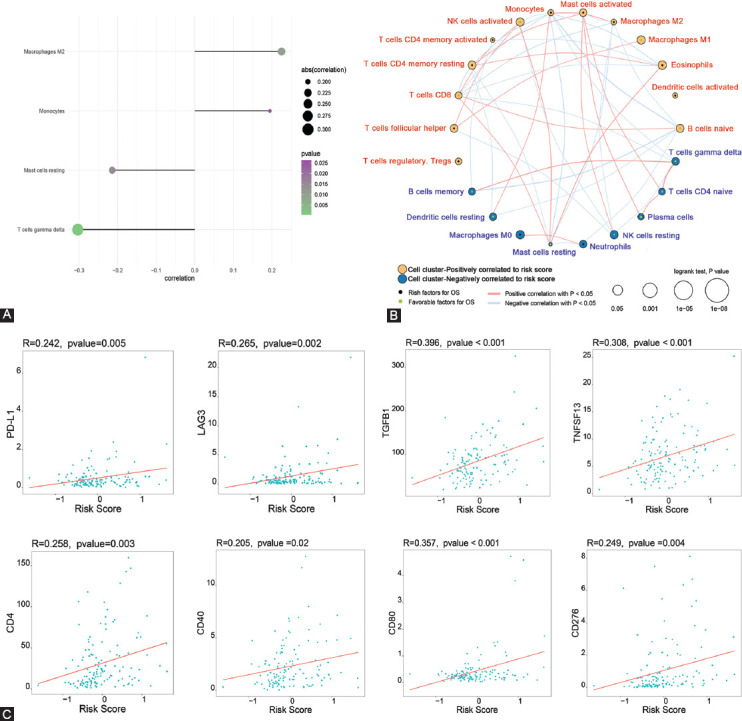
Association between immune status and the 12 FRGs signature. (A) Immune cell component comparisons between low- and high-risk AML subgroups by CIBERSORT. (B) Immune cell infiltration interaction network based on the risk scores and the 22 immune cells. (C) Correlation analysis of the expressions of eight immune checkpoints with 12 FRGs based risk scores. FRGs: Ferroptosis-related genes, AML: Acute myeloid leukemia.

**FIGURE S1:**
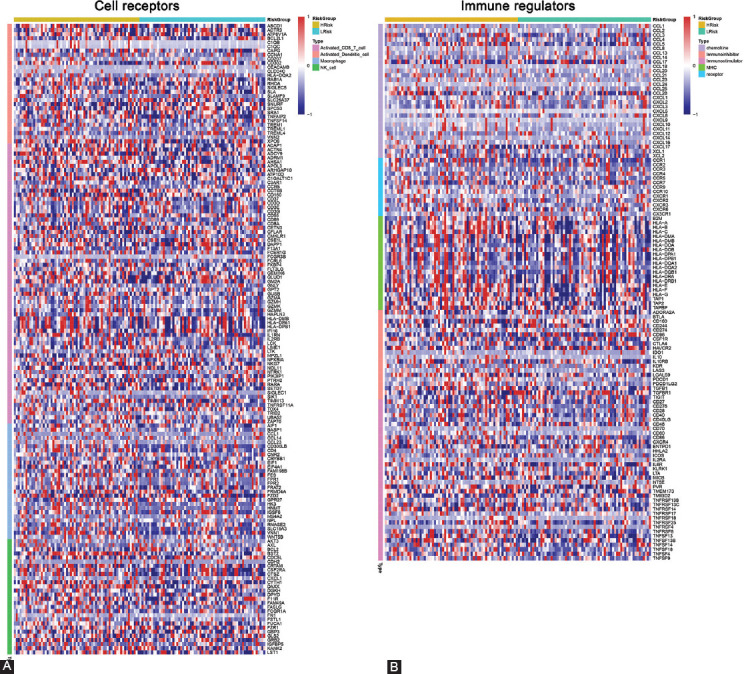
(A and B) Heatmaps of correlation between immune factors and 12 FRGs by the TISIDB database. FRGs: Ferroptosis-related genes.

To study the relationships between risk scores and immunity in the TCGA AML cohort, we assessed correlation between immune cell infiltrations and risk scores using CIBERSORT analysis of the distribution of 22 TIICs in high- versus low-risk AML subgroups. This analysis found that compared to low-risk patient groups, the high-risk group contained a markedly higher proportion of M2 macrophages and monocytes, while resting mast cells and gamma-delta T-cells were low (Figure 7A). Based on the 22 immune cells and risk score, we also established an immune infiltration interaction network (Figure 7B). Next, analysis of Spearman correlations of risk scores and levels of immune-related factors in the TISIDB database, such as immune cell receptor-related genes and immune regulatory genes (chemokines and its receptors, immune inhibitory and stimulatory factors, as well as MHC), revealed that levels of cell receptor-associated genes (EIF4A1, SPCS3, GRB2, BCL2, MPZL1, NOL11, CD302, CFLAR, ATP10D, and CDC5L) were generally upregulated (log2 fold change >2.5) in high-risk group (Figure S1A and Table S4). In addition, some immune regulatory genes showed marked differential expressions (log2 fold change >2.5 or <−2.5) between high- and low-risk groups (B2M, CD96, TAPBP, CD244, TGFBR1, IL2RA, CCR1, HLA-DMA, and HLA-B) (Figure S1B and Table S5). These analyses confirmed that the 12 FRG-based AML risk scores are strongly associated with TIICs infiltration levels and play key roles in tumor immune microenvironment. Correlation assessment of the association between risk score and immune checkpoint-related genes found that PD-L1, LAG3, TGFB1, TNFSF13, CD4, CD40, CD80 (B7-1), and CD276 (B7-H3) positively related to the risk score (Figure 7C). In other words, AML patients with higher risk scores had higher expression levels of these immune checkpoints.

### The association between drug sensitivity and the 12 FRGs signature

**FIGURE 8:**
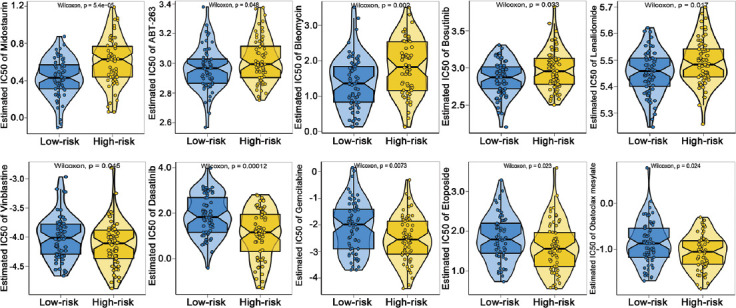
(A-J) Association between risk-related FRGs and drug resistance. Plots denote correlation of risk scores with the IC_50_ of various drugs on AML patients. FRGs: Ferroptosis-related genes, AML: Acute myeloid leukemia.

Based on drug sensitivity data on GDSC, we analyzed the correlation between the 12 FRGs risk score with I_C5_0 of some chemotherapy or targeted drugs used against hematological malignancies. We presumed that a positive correlation between risk score and IC_50_ value would indicate a basis for developing drug resistance in high-risk group patients, while a negative association implies a higher drug sensitivity in these patients. This analysis showed that AML patients in high-risk group had a worse response (higher IC_50_) to Midostaurin, ABT-263, Bleomycin, Bosutinib and Lenalidomide (*p* < 0.05; Figure 8A-E) and were more sensitive (lower IC_50_) to Vinblastine, Dasatinib, Gemcitabine, Etoposide, and Obatoclax mesylate (*p* < 0.05; Figure 8F-J).

## DISCUSSION

According to the latest statistics, 5-year AML survival from 2010 to 2016 is <30% [30]. Most AML patients experience relapse or primary refractory disease, which are hard to treat [4]. Cancer resistance to apoptosis is a major obstacle to successful treatment, resulting in many cancer-associated deaths [31]. Resistance to apoptosis is also a significant hallmark in relapsed/refractory AML [6,7]. Thus, treatments that induce non-apoptotic cell death may improve AML outcomes.

Ferroptosis, a novel form of RCD, is defined by unique morphology, genetics, as well as biochemistry. Since ferroptosis mechanisms differ from apoptosis, it can overcome the low efficacies of apoptosis-initiating drugs. Ferroptosis is mainly induced by iron-dependent enhancement of lipid peroxidation to lethal levels. Several small molecules promote ferroptosis, including erastin and RSL3 [13], and some novel agents can trigger ferroptosis in AML cells [17-21]. These ferroptosis inducers offer alternatives for apoptosis-resistant cases [32]. Ferroptosis is a complex process regulated by various genes [33]. However, the molecular basis of ferroptosis in cancer, including AML, needs further study. Hence, identification of FRG signatures in AML will elucidate on ferroptosis regulatory networks and inform biomarker-based risk stratifications for ferroptosis-associated therapy. Here, we assessed the levels of 103 FRGs in AML patients and their correlation with prognosis. First, a new 12 FRGs prognostic signature was constructed and validated on internal and external cohorts. Based on this model, our functional analyses revealed differential pathways and biological functions between high- and low-risk subgroups, as well as differences in the immune microenvironment and treatment sensitivity.

Using publicly available AML datasets, we for the 1^st^ time show that FRGs can classify patients into high- and low-risk patient subgroups that exhibit marked differences in clinical prognosis and biological features. LASSO regression analysis was used to develop a 12 FRG-based prognostic risk model comprised 10 risk-related genes (GPX4, CD44, FH, CISD1, SESN2, LPCAT3, AIFM2, ACSL5, HSPB1, and SOCS1) and two protective genes (ACSL6 and G3BP1). Thus, AML patients can be classified into subgroups with different risk scores to predict clinical outcomes. Based on function, the 12 FRGs can be grouped into four classes: Lipid metabolism (GPX4, LPCAT3, ACSL5, and ACSL6), antioxidant metabolism (CD44, SESN2, and AIFM2), iron metabolism (CISD1 and HSPB1), and cancer metabolism (SOCS1, FH, and G3BP1) [14,33-36]. GPX4, a central negative regulator of ferroptosis, functions as a lipid repair enzyme that suppresses PL hydroperoxide levels within membranes as well as lipoproteins, inhibiting ferroptosis induction. GPX4 inactivation or degradation induces rapid lipid peroxides accumulation, contributing to ferroptosis [11,12]. GPX4 is elevated in various cancer types where it enhances anti-cancer drug resistance [37]. GPX4 overexpression in AML correlates with poor prognosis [15]. LPCAT3 catalyzes the insertion of arachidonic acid into polyunsaturated fatty acid-containing PLs and can make cells resistant to ferroptosis [38]. LPCAT3 is reported to regulate intestinal stem cell proliferation and enhance tumor formation [39]. ACSL5 and ACSL6 belong to the ACSLs that catalyze activations of long-chain fatty acids. ACSLs deregulation is reported to promote cancer cell proliferation [36] and the activity of ACSL5 and ACSL6 is often enhanced in some solid tumors like colorectal cancer [36]. On the contrary, ACSL6 downregulation significantly correlates with poor patient survival and acts as a tumor suppressor in AML, which is consistent with our findings [40]. Nevertheless, due to a lack of experimental proof, the functional value of ACSL5 and ACSL6 in ferroptosis remains unclear. For antioxidant metabolism, overexpression of the cancer stem cell marker CD44 enhances the stability of SLC7A11, a key regulator of lipid peroxidation, and inhibits ferroptosis in cancer cells [14]. Notably, CD44 is leukemogenic and correlates with poor AML prognosis [41]. SESN2 is a conserved antioxidant that responds to various stresses, including oxidative stress to restore homeostatic balance. SESN2 is reported to protect against iron overload and ferroptosis in liver injury [35]. In the mitochondria, AIFM2 is regarded as a traditional initiator of apoptosis and has been established to be an antioxidant regulator in ferroptosis, independently of mitochondrial function [33]. SESN2 and AIFM2 are responsible for tumor cell survival [33,35], but their exact functions in AML cells ferroptosis remains uncharacterized. Low iron utilization levels can enhance ferroptosis sensitivity. CISD1 may suppress ferroptosis by inhibiting iron uptake by mitochondria [33] and CISD1 inhibition by small molecular ligand NL-1, which is reported to overcome drug resistance and exert anti-leukemic activity in B-cell acute lymphoblastic leukemia [42]. In various cancer cell types, HSPB1, a heat shock protein, is highly induced by erastin therapy. HSPB1 negatively regulates ferroptosis by inhibiting iron uptake [43]. In terms of cancer metabolism, TP53 gene, a well-known tumor suppressor [44], is inactivated or mutated in most human tumors, such as AML. G3BP1 is a TP53 regulator. Interaction between G3BP1 and lncRNA P53RRA traps TP53 in the nucleus, leading to TP53-mediated cell cycle arrest, ferroptosis, as well as apoptosis [45]. It is reported that low G3BP1 expression is related to poor AML survival, highlighting the protective role of G3BP1 [46]. Another TP53 regulator, SOCS1, is required for p53 activation and the regulation of cellular senescence and modulates p53 target genes expression (e.g., SLC7A11) and sensitizes cells to ferroptosis [47]. Interestingly, AML patients with elevated SOCS1 levels have been reported to have low complete remission rates and short OS, indicating that SOCS1 is a poor prognosis predictor of AML [48]. Fumarate hydratase (FH), a TCA cycle enzyme, has been shown to be a tumor suppressor and FH loss of function confers cancer cells resistance to ferroptosis. However, FH expression is often upregulated in tumor cells, including renal cancer and AML cells, which can be explained as FH mutation and dysfunction and needs further investigation [34,49]. The 12 FRGs are highly correlated with tumor prognosis and ferroptosis, which provides a vital theoretical basis for our ferroptosis-based AML risk model. Using GSEA-based GO and KEGG analysis, we identified various BP as enriched in the high-risk group, most of which are ferroptosis related, including glutathione (GSH) metabolic process and PL catabolic process [33].

The notion that immunity promotes or suppresses tumorigenesis is well-accepted. During the development of various cancers, immunosuppressive mechanisms are initiated to avoid anti-tumor immune responses. With increasing immunosuppression, low immunogenic cancer cells are selected, resulting in immune escape [50]. Even though the mechanisms involved in tumor predisposition to ferroptosis are a hot topic in recent years, potential modulation between tumor immunity and ferroptosis remains incompletely understood. Based on the 12 FRGs signature, we performed multiple analyses between different AML risk subgroups and discovered that ferroptosis may be highly associated with tumor immunity. In this study, AML patients with higher risk scores identified by the 12 FRGs signature exhibited higher fractions of M2 macrophages and monocytes. Increased infraction by tumor-associated M2 macrophages and monocytes represents a significant predictor of poor clinical AML outcomes [51]. Moreover, the 12 FRGs based risk score had significant positive correlation with most immune inhibitors (including CD96, CD244, and TGFBR1) and showed significant negative correlation with the immune stimulator IL2RA. These results indicated that ferroptosis and iron metabolism regulate the tumor immune microenvironment and that the poor prognostic outcomes in the high-risk patient subgroup may be as a result of stronger immunosuppression. Immune checkpoint manipulation has recently become a vital, effective immunotherapeutic form [52]. Higher expression of PD-L1, LAG3, TGFB1, TNFSF13, CD4, CD40, CD80, and CD276 was found in the high-risk group, which may enhance AML development and progression, leading to poor prognostic outcomes. Our findings imply that the FRGs risk model may be beneficial to the precision immunotherapy of AML patients in the future, especially those of high-risk groups.

Finally, we explored the association between FRGs base risk scores and drug sensitivities in AML patients. AML patients with higher risk scores were predicted to exert a worse sensitivity against anti-tumor agents including Midostaurin (an FLT3 inhibitor), ABT-263 (a BCL-2 inhibitor), Bleomycin, Bosutinib, and Lenalidomide. Interestingly, those patients seem to be more sensitive to another BCL-2 inhibitor, Obatoclax mesylate, which was demonstrated to show only modest efficacy in the treatment of hematological malignancies including AML, and have not been approved for clinical use yet [53]. Our results also suggested that higher risk patients had a better response to Vinblastine, Dasatinib, Gemcitabine, and Etoposide. Although some of these drugs above are not in clinical use of AML treatment or under investigation, elucidation of the association between the 12 FGRs risk model and drug sensitivity may reveal therapeutic markers for further validation. Through molecular stratification of patients, drug sensitivity data can also optimize clinical trial designs for future successful anti-cancer treatments.

Some limitations of this study are also acknowledged. First, our risk model was constructed and validated using existing public datasets and more prospective data are needed to validate its clinical application. Second, the use of a single hallmark (ferroptosis) for prognostic model construction has some intrinsic weakness. This is because various prognostic genes for AML could have been excluded. In addition, our findings need further confirmation in larger experimental and clinical studies.

## CONCLUSION

We established a novel prognostic model of 12 FRGs in AML. The model successfully divided patients into high- and low-risk patient groups with mean OS as the basis. The underlying mechanisms between 12 FRGs based risk scores and tumor immunity or drug sensitivity in AML were also discussed. We believe that the 12 FRGs have the potential to become a prognostic biomarker that will offer novel insight into AML research and treatment.

## Figures and Tables

**TABLE S1:**
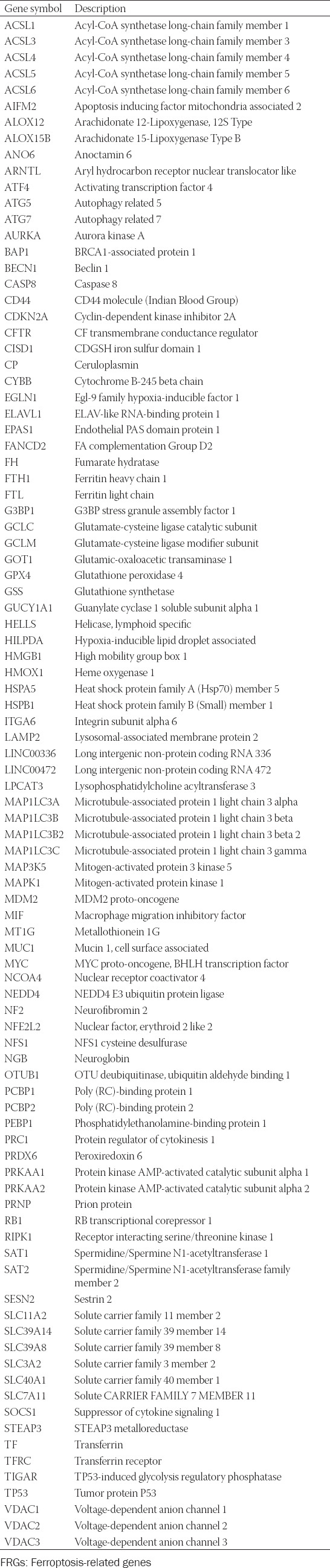
List of the 94 FRGs enrolled in this study

**TABLE S2:**
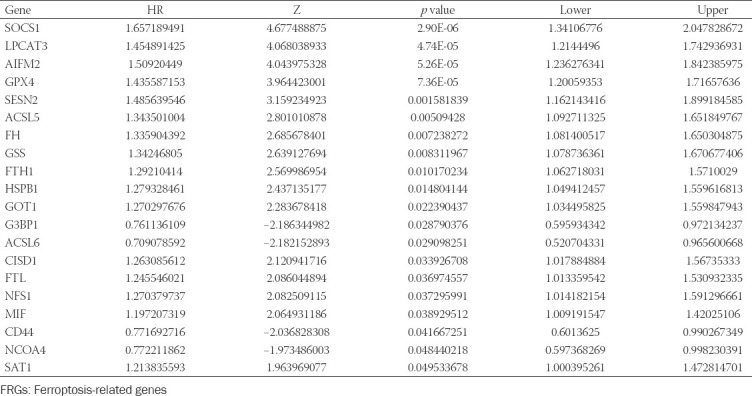
Twenty FRGs with the prognostic values determined by univariate Cox analysis

**TABLE S3:**
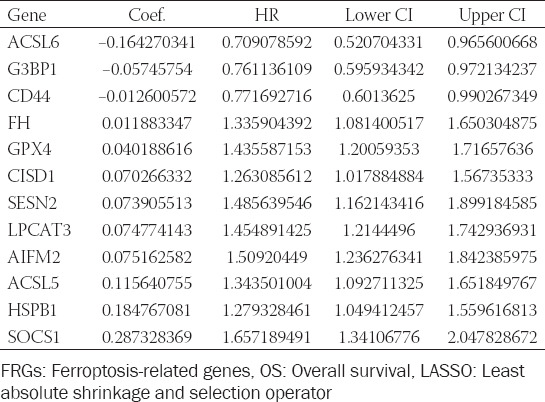
Twelve FRGs signature for OS generated by LASSO Cox regression analysis

**TABLE S4:**
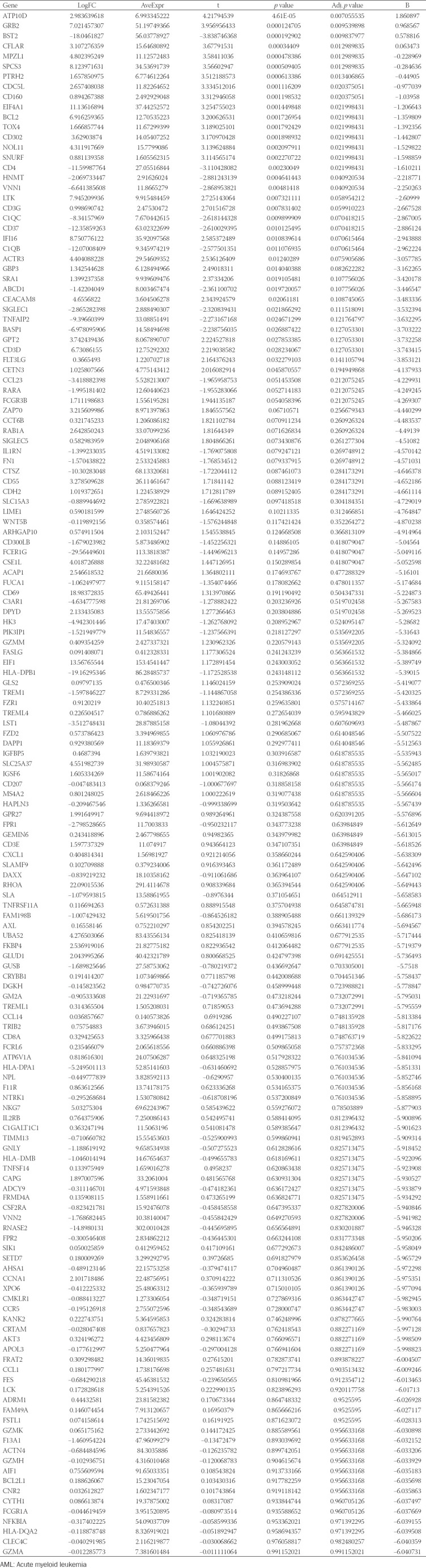
Differential expressions of immune cell receptor-associated genes between low-and high-risk AML group

**TABLE S5:**
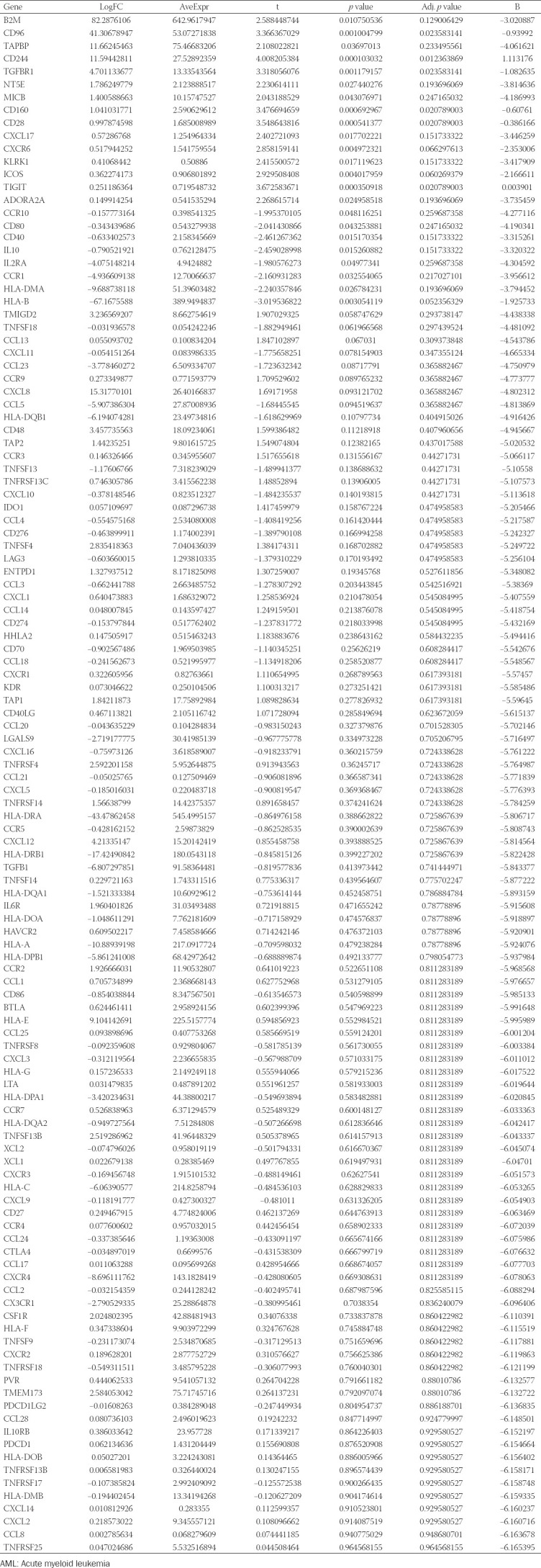
Differential expressions of immune regulatory genes between high-and low-risk AML group
